# Between linkage and return: the engagement strategies of the Knowledge Brazil Program for the scientific diaspora

**DOI:** 10.3389/frma.2026.1854887

**Published:** 2026-09-17

**Authors:** Mariana Luiza Castilho Cavasin, Ana Maria Carneiro

**Affiliations:** 1 Department of Science and Technology Policy, Institute of Geosciences, University of Campinas (UNICAMP), Campinas, Brazil; 2 Center for Public Policy Studies, University of Campinas (UNICAMP), Campinas, Brazil

**Keywords:** Brazil, science, technology and innovation policy, scientific diaspora, state, SWOT analysis

## Abstract

In recent decades, countries of origin have increasingly sought to develop policies to engage with their scientific diaspora. These policy designs typically vary across models of repatriation, linkage, and circulation, aiming to strengthen national science, technology and innovation (STI) systems. This article analyses the design and early implementation of the Knowledge Brazil Program to identify the strategies prioritized by the Brazilian state in engaging with its scientific diaspora. The study is based on document analysis, program microdata, and media sources, including news reports and opinion articles from widely circulated outlets. The findings are synthesized through a SWOT analysis, identifying strengths in the program's design, which incorporates the three core components of diaspora engagement—repatriation, linkage, and circulation; weaknesses in the asymmetry between the program's axes, with a clear emphasis on repatriation; opportunities in the diaspora's willingness to contribute to the national STI system; and threats related to concerns regarding the absorption of researchers into academia after the program's completion.

## 1 Introduction

Science has historically been characterized by an international dimension. A study by Olechnicka et al. (2019), based on international collaboration measured through co-authorship in publications, shows that since the early twentieth century, and with increasing intensity in recent decades, there has been a steady rise in international scientific collaboration. According to the authors, this trend is associated with growing global integration and the increasing interdependence of societies. These processes have been facilitated by the liberalization of flows of goods and services, technological advances over recent decades, and the growing recognition of knowledge as a key asset for societies, all of which have reshaped academic practices and made the scientific community more interconnected (Olechnicka et al., 2019; Guzmán-Valenzuela, 2025).

International collaboration has undergone substantial transformations, both quantitatively—reflected in the growth of co-authored publications, international research projects, jointly held patents, collaborative events, and shared research infrastructure—and qualitatively, as large-scale collaboration has enabled scientific achievements that would not be possible otherwise (Olechnicka et al., 2019). These changes affect not only university-based academics but also researchers working in industrial and corporate laboratories (Olechnicka et al., 2019).

However, the authors emphasize that the dynamics of research internationalization are unevenly distributed across the world, following a center-periphery pattern: “core countries, due to the availability of resources and accumulated academic prestige, are able to attract talented researchers from peripheral countries” (Olechnicka et al., 2019, p. 103). This global dynamic has created and reinforced asymmetries in the circulation and concentration of knowledge (Marginson, 2022) because the spaces in which knowledge is distributed, consumed, and produced are shaped by unequal power relations (Paasi, 2015). For example, the hierachy of spaces of writing and the valorization of English as a lingua franca reinforce the tendency for hegemonic knowledge to circulate from the center to the peripheries (Paasi, 2015).

One of the central aspects of the internationalization of science is the mobility of academics. Studies spanning the twentieth and twenty-first centuries indicate a growing trend in the mobility of highly skilled individuals (Brum, 2024; Ciumasu, 2010; Olechnicka et al., 2019; Tejada, 2012). According to Ackers (2004), employment trajectories, career progression, and international experience are closely intertwined for those working in scientific research. In this context, researcher mobility is partly driven by career opportunities, as well as by the availability of resources and infrastructure in destination countries. Academic mobility policies may also be associated with diplomacy between countries, international partnerships, and cooperation agreements that may involve funding mechanisms (Finardi et al., 2020). This intercountry dynamic is predominantly oriented towards linkages with countries in the Global North (Finardi et al., 2020).

From the perspective of peripheral countries, this mobility is particularly significant, as access to the science, technology, and innovation (STI) systems of core countries can create opportunities for academic and industrial development (Pandey et al., 2022). It is within this framework that the issue of engagement with scientific diasporas emerges. The term scientific diaspora refers to the migration of highly skilled professionals, particularly scientists, who carry out research activities and reside outside their countries of origin while maintaining ties with their home country (Carneiro et al., 2020). These actors can influence international cooperation by acting as bridges for the exchange and circulation of knowledge, thereby contributing to the development of STI systems in countries of origin, destination countries, and beyond (Tejada, 2012).

Early approaches to the movement of highly skilled individuals were framed within the “brain drain” paradigm, which described flows from developing to developed countries. From this perspective, countries of origin experience a loss of qualified human capital, whereas destination countries benefit from “brain gain,” thereby further strengthening their human capital and scientific development (Brum, 2024; Meyer, 2003).

Since the 1990s, several scholars have advanced a new perspective on the mobility of highly skilled individuals, viewing it as a circular rather than unidirectional process (Carneiro et al., 2022; Ciumasu, 2010). The emergence of so-called diaspora networks challenges the traditional concept of brain drain by reframing it as a process of knowledge circulation, whereby the loss of talent is transformed into a remote and accessible asset that contributes not only to the country of origin but also to other countries (Meyer and Wattiaux, 2006; Balbachevsky and Couto e Silva, 2011; Tejada, 2012). This dynamic has shifted the focus from a return-based approach to a linkage-oriented perspective, in which technical and institutional connections are central, and enabling structures are required in both origin and destination countries to sustain cooperation (Meyer and Wattiaux, 2006; Tejada, 2012). In this context, Ciumasu (2010) emphasizes that international mobility should be included in the policy agenda to develop sustainable collaborative strategies.

In Brazil, initiatives aimed at engaging the scientific diaspora are relatively recent. Brum (2024) argues that, until around 2010, talent migration was predominantly interpreted through the lens of brain drain. Carneiro et al. (2022) note that the discourse on the international mobility of Brazilian researchers has fluctuated over time between positive and negative interpretations. These shifts can be attributed to political, economic, and social instability at different moments. Nevertheless, the brain drain perspective has remained a recurring narrative, although with varying intensity.

From 2010 onwards, policies began to emerge that recognized the strategic role of the scientific diaspora. Brum (2024) notes that the Brazilian state formally adopted a diaspora-oriented approach in 2012 with the launch of the Rede Diáspora Brasil project (2012–2016), which was part of the international innovation agenda of the Brazilian Agency for Industrial Development (ABDI). The initiative aimed to strengthen linkages between Brazilian professionals abroad and Brazil through the promotion of partnerships and the development of collaborative networks. Another strategy designed to promote collaborative networks and engagement with Brazilians abroad is the Innovation Diplomacy Program (PDI), launched in 2017 by the Ministry of Foreign Affairs (MRE). Among the shared objectives of Rede Diáspora Brasil and the PDI are mobilizing the scientific diaspora, fostering partnerships, sharing experiences, supporting public policy formulation, strengthening connections and dialogue with the diaspora, and expanding collaborative networks (Brasil, 2026a.; Brum, 2024; Carneiro et al., 2020).

Although the initiatives mentioned indicate a shift in discourse towards recognizing the diaspora as a strategic asset, more assertive policies focused on long-term engagement are still needed. Carneiro et al. (2020) emphasize that the strength of the linkage between the diaspora and its country of origin depends on the design of public policies that foster sustained relationships and commitments for interaction between countries.

In 2024, the Brazilian federal government launched the Knowledge Brazil Program, a new initiative aimed at engaging the scientific diaspora. The program is coordinated by the Ministry of Science, Technology and Innovation (MCTI) and implemented by the National Council for Scientific and Technological Development (CNPq), with funding from the National Fund for Scientific and Technological Development (FNDCT), the country's main public instrument for financing research, science, and innovation. The program aims to strengthen national scientific capacity and foster cooperation with the Brazilian scientific diaspora.

In this context, this article analyses the design and early implementation of the Knowledge Brazil Program to understand how this policy is positioned within the scholarly debate on scientific diaspora engagement and to identify the types of strategies prioritized by the Brazilian state in engaging with its diaspora. This study is justified by its systematic and analytical approach to a recent Brazilian policy initiative. It also holds international relevance by providing empirical evidence on the governance of multiple actors involved in the formulation and implementation of scientific diaspora engagement policies, particularly in countries seeking to strengthen their science, technology and innovation (STI) capacities. To this end, the study adopts a methodology that combines document analysis of official program calls and microdata with an examination of media coverage, including news reports and opinion articles. The findings are synthesized through a Strengths, Weaknesses, Opportunities, and Threats (SWOT) analysis. The use of SWOT enables policymakers to diagnose internal and external environments in order to establish strategies for the formulation and implementation of programs and policies. The remainder of this article is organized into four sections. The next section provides a brief overview of strategies for engaging scientific diasporas. The third section outlines the methodology and analytical approach adopted in the study. The fourth section presents and discusses the results, including a detailed description of the Knowledge Brazil Program, and synthesizes the findings through a SWOT analysis. Finally, the last section presents the conclusions, including the study's limitations and directions for future research.

## 2 Strategies for engaging with the scientific diaspora

This section examines initiatives aimed at engaging scientific diasporas in selected countries in Latin America, as well as in China and India. Its purpose is to provide an overview of the types of strategies adopted by these countries, which will subsequently serve as a basis for the analysis of the Knowledge Brazil Program.

According to Shin and Moon (2018), countries adopt different strategies in response to the international mobility of highly skilled individuals. These strategies often provide the foundation for initiatives designed to engage scientific diasporas. The authors identify four main approaches: (1) *brain train and retention*, which focuses on the education and training of highly qualified professionals, combined with policies aimed at retaining them domestically and discouraging outward mobility; (2) *brain gain*, which is associated with talent attraction and involves the recruitment or training of skilled professionals from abroad; (3) *brain circulation*, whereby countries send individuals abroad for training with the expectation that they will later return, thereby promoting the circulation of talent and supporting repatriation strategies; and (4) *brain linkage*, which relies on mechanisms of interaction with nationals who choose not to return, including the formation of networks, the development of scientific collaborations, and other institutional arrangements.

A substantial body of literature has examined policies for engaging scientific diasporas across different national contexts (Bonilla et al., 2022; Brum, 2024; Ciumasu, 2010; Kramer and Zent, 2019; Pandey et al., 2022; Shin and Moon, 2018; Tejada, 2008). Shin and Moon (2018), for instance, analyze the cases of China, India, Taiwan, and South Korea. In all four cases, examined from the 1950s onwards, the United States was the main destination country for members of the scientific and highly skilled diaspora. The strategies adopted by these governments to leverage the potential of their diasporas, particularly from the 1990s onwards, emerged in a context of economic growth and involved investments in industrial development and in the expansion of research and development (R&D) institutions, with the aim of attracting and retaining expatriate researchers.

The case of China illustrates the gradual implementation of return-oriented incentives. Shin and Moon (2018) show that, due to limited state resources to support permanent return, the Chinese government strategically encouraged short-term returns, enabling expatriates to establish linkages that could be sustained after their departure. These initiatives therefore combined elements of repatriation, circulation, and linkage strategies.

Kramer and Zent (2019) argue that, in general, internationally mobile researchers tend to be more productive than non-mobile researchers. One of the outcomes of the initiatives discussed in the literature is the formation of global collaboration networks, which have contributed to processes of industrial modernization in countries of origin. For example, engineers based in Silicon Valley became actively connected with returnees, facilitating the exchange of knowledge and resources in both China and India (Shin and Moon, 2018). Another outcome was the development of research infrastructure in countries of origin, supported by government efforts to attract these professionals. One example is the return of Indian researchers who contributed to the establishment of the IBM India Research Laboratory, founded in 1998 (Shin and Moon, 2018).

In Latin America, several policies have also been developed to engage scientific diasporas (Bonilla et al., 2022; Brum, 2024; Tejada, 2008). Colombia's Red Caldas, or Caldas Network, established in 1991, is an emblematic case and is widely regarded as a pioneering initiative in the region (Bonilla et al., 2022). The policy aimed to strengthen scientific capacity in Colombia by fostering linkages between Colombian nationals residing abroad and domestic research institutions. According to Bonilla et al. (2022), the activities of Red Caldas were organized around four main strategies: cooperation and the development of joint projects; human resource training and researcher return; circulation and international mobility; and scientific communication and dissemination. While Colombian institutions created a favorable environment for interaction, the motivation and mobilization capacity of diaspora researchers were key factors in making the network effective (Tejada, 2008).

Another relevant initiative is Converciencia, a Guatemalan policy for engaging the scientific diaspora. Launched in 2005 by Guatemala's National Secretariat of Science and Technology (Senacyt), the policy aims to connect Guatemalan scientists residing abroad with their country of origin, involving the industrial sector, academia, and society more broadly (Bonilla et al., 2022). The policy encompasses three main groups of participants: science and technology (S&T) policy actors; the scientific community, including members of the Guatemalan scientific diaspora; and host institutions, particularly universities and research institutes. Within the scope of Converciencia, the RedCTI network was established to foster coordination among institutions in Guatemala, engage Guatemalan scientists abroad, and promote scientific communication and knowledge transfer for national development. According to Bonilla et al. (2022, p. 5), “RedCTI constitutes one of the main initiatives supported by Senacyt to promote science and technology for national development.”

In Argentina, the RAICES Program (Network of Argentine Researchers and Scientists Abroad), was created in 2003 and became law in 2008. According to Bonilla et al. (2022), the objectives of this policy include both the permanent and temporary repatriation of Argentinians residing abroad, as well as the strengthening of linkages between domestic researchers and members of the diaspora. Repatriation is facilitated through coordination with the National Scientific and Technical Research Council (CONICET), which integrates returning researchers into its Scientific and Technological Research Career through the CIC-EXT modality, designed for researchers coming from abroad. This mechanism provides employment opportunities in Argentine universities and strategic sectors, thereby helping to attract and retain scientists who wish to return to the country (CONICET, 2021).

Linkage-oriented initiatives include the development of networks connecting Argentine researchers based in the country and abroad; the international dissemination of national scientific and technological activities; improved access to information on highly qualified Argentinians residing abroad; and the integration of diaspora researchers into scientific, technological, and innovation activities promoted by the national government (Argentina, 2019). A third strategy focuses on circulation and targets researchers who wish to return temporarily to Argentina. This is operationalized through the RAICES subprogram Milstein, which promotes linkages with the local scientific and technological environment by funding short-term stays of 1 to 3 months (Argentina, 2019).

In Brazil, as noted earlier, the Rede Diáspora Brasil and the PDI share common objectives related to fostering collaboration and engagement with Brazilians residing abroad (Brum, 2024; Brasil, 2026a). These initiatives can be understood as linkage-based strategies aimed at strengthening connections between the country of origin and its scientific diaspora, with an emphasis on the development of collaborative networks.

Table 1 summarizes the types of strategies adopted by different countries to engage with the scientific diaspora.

**Table 1:** Types of strategies for engaging with the scientific diaspora.

Typology of strategies	Description	Examples
Repatriation-focused	Incentives for the return of the diaspora through attraction and retention in industrial or academic sectors	Chinese government program (implemented gradually)
Circulation	Incentives for short-term mobility to countries of origin	- Milstein subprogram in Argentina (within the RAICES policy) – Chinese government program (as part of a repatriation strategy)
Linkage	Collaboration without the need for return	– Rede Diáspora Brasil – Innovation Diplomacy Program (PDI) (Brazil) – Converciencia (Guatemala)
Comprehensive (repatriation, circulation, linkage)	A combined model integrating repatriation, circulation, and linkage strategies	– RAICES (Argentina) – Caldas Network (Colombia)

Authors' own elaboration based on Shin and Moon (2018), Brum (2024), Brasil (2026a), Bonilla et al. (2022), Tejada, 2008, CONICET (2021) and Argentina (2019).

## 3 Methodology

The study adopted a qualitative approach (Flick, 2009), with a descriptive and explanatory orientation, and was based on document analysis and secondary data. The research was conducted in five stages.

### 3.1 Document analysis of the Knowledge Brazil Program

The first stage consisted of analyzing the program's documents (Godoy, 1995). This stage aimed to examine how the program was designed and implemented as a public policy, as well as to support the construction of the SWOT analysis by identifying internal factors—strengths and weaknesses. It was based on official ordinances, calls for proposals, and their respective results published by the National Council for Scientific and Technological Development (CNPq) and the Brazilian Innovation Agency (FINEP). This stage was divided into two phases. In the first phase, documents announcing and establishing the program were analyzed, including official ordinances and calls for proposals related to the Knowledge Brazil Program. The analysis sought to describe the program's policy design, its main objectives, the coordination among the actors involved, as well as its sources of funding and the benefits provided.

In the second phase, the program's implementation was analyzed based on the results of the calls for proposals, considering the relationship between the estimated number of projects to be funded, the number of submitted and approved proposals, the allocated funding amounts, and the regional distribution of projects.

Table 2 summarizes all documents used in this stage, including their publication dates and access links.

**Table 2:** Documents used for document analysis.

Document	Period	Links
CNPq Ordinance No. 1,708, of 9 April 2024	Published in the Official Gazette (*Diário Oficial da União*) on 12 April 2024	http://memoria2.cnpq.br/web/guest/view/-/journal_content/56_INSTANCE_0oED/10157/21594631
CNPq Ordinance No. 2,565, of 12 December 2025	Published in the Official Gazette (*Diário Oficial da União*) on 15 December 2025	http://memoria2.cnpq.br/web/guest/view/-/journal_content/56_INSTANCE_0oED/10157/22997482
Call for Proposals CNPq/MCTI/FNDCT No. 21/2024 – Knowledge Brazil Program – Attraction and Retention of Talent	Application period: 29 July 2024 to 1 October 2024	http://memoria2.cnpq.br/web/guest/chamadas-publicas?p_p_id=resultadosportlet_WAR_resultadoscnpqportlet_INSTANCE_0ZaM&filtro=abertas&detalha=chamadaDivulgada&idDivulgacao=12345
Final Results of Call for Proposals CNPq/MCTI/FNDCT No. 21/2024 – Knowledge Brazil Program – Attraction and Retention of Talent	3 June 2025	http://memoria2.cnpq.br/web/guest/chamadas-publicas?p_p_id=resultadosportlet_WAR_resultadoscnpqportlet_INSTANCE_0ZaM&filtro=abertas&detalha=chamadaDivulgada&idDivulgacao=12345
Call for Proposals CNPq/MCTI/FNDCT No. 22/2024 – Knowledge Brazil Program – Support for Network Projects with Brazilian Researchers Abroad	Application period: 29 July 2024 to 13 September 2024	http://memoria2.cnpq.br/web/guest/chamadas-publicas?p_p_id=resultadosportlet_WAR_resultadoscnpqportlet_INSTANCE_0ZaM&id=47-2484-9468&detalha=chamadaDetalhada&filtro=abertas
Final Results of Call for Proposals CNPq/MCTI/FNDCT No. 22/2024 – Knowledge Brazil Program – Support for Network Projects with Brazilian Researchers Abroad	24 December 2024	http://memoria2.cnpq.br/web/guest/chamadas-publicas?p_p_id=resultadosportlet_WAR_resultadoscnpqportlet_INSTANCE_0ZaM&id=47-2484-9468&detalha=chamadaDetalhada&filtro=abertas
Public Call MCTI/FINEP/FNDCT – Economic Subsidy for Innovation (Continuous Flow) – Knowledge Brazil	Launched on 7 August 2024 (continuous flow)	https://www.finep.gov.br/chamadas-publicas/chamadapublica/745
Partial Results of the Public Call MCTI/FINEP/FNDCT – Economic Subsidy for Innovation (Continuous Flow) – Knowledge Brazil	31 December 2025	http://finep.gov.br/images/chamadas-publicas/2026/07_01_2026_resultado_parcial_Conhecimento_Brasil_dezembro_2025.pdf

Authors' own elaboration based on CNPq (2024a,b,c, 2025a); FINEP (2024a).

### 3.2 Secondary data analysis

The second stage involved the analysis of secondary data. Microdata from the results of the calls for proposals, available on the CNPq open data platform, were used. The objective was to identify the researchers funded under Call CNPq/MCTI/FNDCT No. 21/2024 – Knowledge Brazil Program – Attraction and Retention of Talent, hereby Call “Attraction and Retention of Talent”.

The data were extracted from the CNPq funding dashboard, available at: http://www.bi.cnpq.br/painel/mapa-fomento-cti/. This database was selected because it is the official data source with publicly available information on the program. Data on grants and fellowships under the Knowledge Brazil Program were downloaded as an Excel spreadsheet in October 2025. This dataset combined information from both program calls (21/2024 and 22/2024) and was structured into 1,165 rows and 18 columns, including the following variables: Lattes ID; beneficiary name; beneficiary CPF (partially anonymized); country; region; state; city; funding line; major field; field; subfield; modality code; modality name; call title; funding program name; institution name; number of grants; and number of fellowships. A descriptive analysis of the collected data was conducted to organize and characterize the observed features of the dataset. The results are presented through charts and tables.

As the objective of this stage was to examine only Call Attraction and Retention of Talent, the dataset was filtered using the criterion “Call CNPq/MCTI/FNDCT No. 21/2024 – Knowledge Brazil Program – Attraction and Retention of Talent.” This process generated a new dataset containing only the researchers funded under the Attraction and Retention of Talent call. As a result, a total of 528 researchers were identified using the variables “beneficiary name” and “Lattes ID” extracted from the CNPq data platform.

### 3.3 Analysis of Lattes CVs

The third stage complemented the second by incorporating data from Lattes CVs. Its objective was to map the profile of fellows benefited from the Call Attraction and Retention of Talent.

The Lattes CV is part of the Lattes Platform, a national database of the academic trajectories of Brazilian researchers. Created and maintained by CNPq, it aims to systematically compile information such as academic background, professional experience, scientific publications, conference participation, language skills, and other relevant data. It is an essential document in Brazilian academic life, serving as the primary curricular basis for evaluating applications from students and researchers in public agencies (Brasil, 2025). The system's reliability has made it the national reference for documenting academic and research trajectories.

The information was manually collected on an individual basis through direct consultation of each Lattes CV between October and December 2025. Four types of information were collected:

1. Highest level of qualification: classified as undergraduate, master's degree or doctoral degree;
2. Year of highest level qualification: used as an indicator of career stage and time since completion;
3. Academic/professional affiliation in 2024: The year 2024 was selected as a reference point to identify researchers' academic and professional affiliations at the time of application to the program call. Affiliations were determined based on information reported in the “summary” and “professional experience” sections of the Lattes CVs. For analytical purposes, the categories “postdoctoral fellow,” “postdoctoral researcher,” and “postdoctoral internship” were standardized as “postdoctoral researcher.” Affiliations were then classified into Postdoctoral researchers or Other fixed-term contract positions (e.g. fellows, visiting researchers/professors, invited researchers/professors, temporary lecturers, and collaborating researchers) and permanent positions (e.g. civil servant researchers, assistant professors, associate professors, full professors, and entrepreneurs).
4. Country of affiliation in 2024: This variable was central to assessing the geographical positioning of researchers at the time of application to the program call. Country information was identified using both the “professional experience” section—given that it includes the start and end dates of activities—and the “professional address” section of the Lattes CVs.

Data were tabulated using Excel's pivot table function. This stage enabled the characterization of the beneficiaries' profile within the Attraction and Retention of Talent policy.

The spreadsheet containing all data is available in Supplementary material.

### 3.4 Document analysis of media materials

The fourth stage consisted of analyzing media sources. The purpose of using these materials was to examine the program's reception within the academic community in Brazil, identifying perceptions, criticisms, and support for the policy. These perceptions informed the construction of the SWOT matrix, particularly regarding external factors—opportunities and threats.

To this end, a survey of news coverage was conducted, including specialized media and mainstream press, across digital news platforms and institutional websites such as *Jornal da USP, Jornal da Unesp, Jornal da Unicamp, Correio do Estado, Estadão, Agência Bori*, news released by CNPq and YouTube videos, among others. The search for news articles was carried out using descriptors such as “Attraction and Retention of Talent Program–Knowledge Brazil,” “CNPq repatriation,” and “Knowledge Brazil Program.” The document analysis was temporally bounded between May 2024 and June 2025, corresponding to the phases of announcement, launch, and early implementation of the program.

The analysis focused on identifying discursive patterns related to perceptions of the program's sustainability, budgetary adequacy, and expectations for its outcomes. In this way, the media analysis contributed to linking the program's formal design to the dynamics of perception and reception within the academic field.

### 3.5 Construction of the SWOT matrix

The fifth stage involved synthesizing the results of the previous stages into a SWOT matrix.

Traditionally used for managerial analysis in private companies, this tool has also been adopted as a methodological approach for analyzing organizations and public policies by identifying strengths, weaknesses, opportunities, and threats across their internal and external environments (Paiva and Guerra, 2021). This tool has recently been used to evaluate public policies for engaging with the scientific diaspora. In the study by Bonilla et al. (2022), the data collected by the authors were synthesized within a SWOT framework, highlighting strengths, weaknesses, opportunities, and threats in the planning, organization, implementation, monitoring, and perceived outcomes of the Converciencia policy.

In this study, the operationalization of the categories of the SWOT Matrix was inspired by the ex-ante evaluation guide developed by the Institute for Applied Economic Research (IPEA, 2018). Internal policy factors were identified primarily through document analysis of official program documents, analysis of secondary data from CNPq, and examination of Lattes CVs. These factors were classified as strengths or weaknesses based on the program's design and implementation, reflecting its potentialities and limitations.

Subsequently, external factors affecting the policy were identified primarily through document analysis of media sources. These factors were classified as opportunities or threats depending on whether they represented favorable conditions for the program's implementation and consolidation or external risks that could limit its chances of success. The analytical procedures were peer-reviewed by the two authors of this manuscript.

## 4 Results and discussion

This section presents the Knowledge Brazil Program, including its announcement and design, expectations, results, and the perceptions of the academic community. All material is systematized in the SWOT Matrix.

### 4.1 Knowledge Brazil Program

Before proceeding with this subsection, it is important to note that no systematic documents were identified indicating whether a formal diagnostic process informed the formulation of the Knowledge Brazil Program. This section is therefore based on statements reported in the media, including interviews with officials from the Ministry of Science, Technology and Innovation (MCTI) and CNPq. According to these actors, the Knowledge Brazil Program represented a shift in the way the country approaches its scientific diaspora, recognizing it as a strategic asset for national scientific development.

In an interview with the news and opinion website *Portal Vermelho* in June 2025, the Scientific Director of CNPq, Olival Freire Jr., stated that, in his view,

The Brazilian state had, until then, largely ignored the scientific diaspora. […] It was only under this government [Luiz Inacio Lula da Silva, third mandate] that the existence of thousands of Brazilian researchers working abroad was recognized as a strategic asset—rather than an administrative problem […] The treatment of the Brazilian scientific diaspora thus became a matter of state concern (Xavier, 2025).

Furthermore, the Scientific Director of CNPq described the creation of the Knowledge Brazil Program as a “structural policy in science and technology,” operating through “repatriation as a state policy” (Xavier, 2025). According to CNPq President Ricardo Galvão, in an interview with the newspaper *Estadão* in April 2024, the program sought to attract “leading researchers” and “people with more experience” (Jansen, 2024).

CNPq's Scientific Director stated that the diagnosis underpinning the need for the program did not result from a structured field study, but rather from active listening during meetings with networks of scientists abroad and from visits conducted by the Minister of Science, Technology and Innovation, Luciana Santos (Xavier, 2025). The meeting took place in Oxford with Brazilian researchers funded by CNPq. According to the Minister, “It was important to discuss how the Brazilian diaspora can contribute to national development strategies and to international scientific and technological cooperation” (Brasil, 2023). Among the topics discussed in this meeting were the possibilities of strengthening linkages between research groups of Brazilians abroad and researchers based in Brazil (Brasil, 2023).

Freire Jr. further explains that, at this diagnostic stage, based on active listening, three target groups were identified for the program: (1) those who wish to return, that is, researchers seeking opportunities to settle in Brazil; (2) those who wish to collaborate without returning, that is, researchers interested in engaging in joint projects and collaborative networks; and (3) those with legal issues who seek to regularize their status by contributing to national science and technology (Xavier, 2025).

With regard to legal issues, it is worth noting that in 2023 CAPES and CNPq issued ordinances introducing significant changes to the rules on contract novation. This measure aims to regularize the status of former scholarship holders with pending return obligations, allowing contribution through novation as an alternative to the mandatory return to and residence in Brazil after completion of the fellowship, for a period equivalent to the duration of the fellowship. In this context, the reform recognizes that engagement with Brazilian researchers abroad can be beneficial to national science, and that the development of research networks and the exchange of knowledge constitute meaningful forms of contribution (CAPES, 2023; CNPq, 2023). However, one limitation of this ordinance is that it does not apply to former scholarship holders who had already signed a debt acknowledgment agreement or who are already registered as having outstanding debts with the federal government (CAPES, 2023; CNPq, 2023). In such cases, participation in the Knowledge Brazil Program could serve as a novation of the obligation, potentially replacing the pecuniary debt (Xavier, 2025).

In 2024, the federal government launched what was initially named the Talent Repatriation Program–Knowledge Brazil (CNPq, 2024d). Implemented by the National Council for Scientific and Technological Development (CNPq) and the Brazilian Innovation Agency (FINEP), this initiative is one of the ten priority actions funded by the National Fund for Scientific and Technological Development (FNDCT) under the National Strategy for Science, Technology and Innovation 2023–2030.

According to CNPq (2024d) and the Minister of Science, Technology and Innovation, Luciana Santos, in an interview on *Bom Dia Ministra* broadcast on the YouTube channel TV 247 (Melo, 2024), the program marks the resumption of investment in science, technology and innovation (STI) in Brazil after a period of budget constraints and funding cuts in the sector. The program was established by CNPq Ordinance No. 1,708 of 9 April 2024 (CNPq, 2024c) and was subsequently amended by CNPq Ordinance No. 2,565 of 12 December 2025 (CNPq, 2025a). According to CNPq (2024d), the initiative aims to encourage the return of researchers residing abroad by offering opportunities either to relocate to Brazil or to strengthen linkages with universities and companies.

It is also worth noting that the Ministry of Foreign Affairs (MRE), in conjunction with the Innovation Diplomacy Program (PDI), played a strategic role in promoting the program through Brazilian missions abroad and in fostering connections with the diaspora. Initial announcements and documents were disseminated through embassies and consulates before publication on the CNPq website.

Brazilian researchers residing abroad will have the opportunity to return to the country to work in universities and companies through the Talent Repatriation Program – Knowledge Brazil, which is set to be launched in May, with two main components: one focused on the attraction and retention of professionals, and another aimed at the development of international scientific cooperation networks (Brasil, 2024).

It is worth noting that the name “Talent Repatriation Program–Knowledge Brazil” underwent a transition over time. Early documents circulated by Brazilian embassies and the initial announcement by CNPq adopted this designation, with a clear emphasis on repatriation. Subsequently, the program gradually came to be identified simply as the “Knowledge Brazil Program”.

In 2024, three calls were launched, two implemented by CNPq and one by FINEP. Within the framework of the program, a new initiative was also launched in 2026, entitled the Program for Supporting the Retention of PhD Holders in Brazil–Knowledge Brazil (PROFIX-CB). This initiative aims to promote the retention of PhD holders in strategic areas of science, technology and innovation across Brazilian states and the Federal District, in partnership with State Research Funding Agencies (FAPs) (CNPq, 2026a). This new call, however, is not the focus of the present study.

Within the scope of CNPq, the program was structured into two components. The first component entitled “Attraction and Retention of Talent,” and implemented through Call No. 21/2024, focuses on funding research projects led by Brazilian researchers, residing in Brazil, who completed their PhD or postdoctoral training abroad from 2019 onwards, as well as by Brazilian master's and doctoral degree holders residing abroad who intend to return to conduct research in Brazil (CNPq, 2024a).

The program envisaged funding up to 1,000 projects across two distinct lines, according to applicants' profiles (CNPq, 2024a), as presented in Table 3.

**Table 3:** Program lines under Call CNPq/MCTI/FNDCT No. 21/2024 – Knowledge Brazil Program–Attraction and Retention of Talent.

Line 1	Line 2
Projects submitted by PhD holders (or candidates in the final stage of their doctoral studies, with defense expected by December 2024) intending to carry out their projects in Scientific, Technological and Innovation Institutions (STIs), public or private Higher Education Institutions, or public and private companies engaged in science and technology activities.	Projects submitted by representatives of public or private companies for the recruitment of master's or doctoral degree holders to develop scientific or technological research projects. Applicants under Line 2 must hold a higher education degree, with no requirement for a stricto sensu postgraduate qualification.

Authors' own elaboration based on Call CNPq/MCTI/FNDCT No. 21/2024 – Knowledge Brazil Program – Attraction and Retention of Talent (CNPq, 2024a).

In this context, for master's degree holders, proposals had to be submitted by a legal representative of the host company, whether public or private, where the project would be carried out. This representative would serve as the project coordinator. Projects submitted under Line 2 were allowed to request more than one fellowship per project (CNPq, 2024a).

In the case of doctoral degree holders, in addition to the possibility of submission through a legal representative of a company, applicants could also submit their own proposals. In such cases, they were required to indicate the scientific, technological, and innovation institution (STI institution) where the project would be carried out, which could include public or private higher education institutions, or public or private companies engaged in science and technology activities. It is noteworthy that formal approval from both the host institutions and the designated team members was required (CNPq, 2024a). Across both lines, applicants were allowed to submit only one proposal (CNPq, 2024a).

Under this call, the program relied on enhanced benefits to attract talent among selected master's and doctoral degree holders. These benefits are presented in Table 4.

**Table 4:** Benefits provided under Call CNPq/MCTI/FNDCT No. 21/2024 – Knowledge Brazil Program–Attraction and Retention of Talent.

Type of benefit	Benefits for Master's degree holders	Benefits for Doctoral degree holders
Fellowship	BRL 10,000 per month	BRL 13,000 per month
Research grant	Up to BRL 400,000	Up to BRL 400,000
Installation allowance	Equivalent to 1 monthly payment, granted in the first month of the project	Equivalent to 1 monthly payment, granted in the first month of the project
Health allowance	Equivalent to 1 monthly payment, granted at the beginning of each 12-month period	Equivalent to 1 monthly payment, granted at the beginning of each 12-month period
Social security contribution allowance	BRL 1,000 per month for contributions to the National Social Security Institute (INSS), equivalent to self-employed contributions	BRL 1,000 per month for contributions to the National Social Security Institute (INSS), equivalent to self-employed contributions
Airfare	Reimbursement of return airfare to Brazil for the project coordinator, including immediate family members (spouse and children), applicable to researchers residing abroad at the time of project approval	Reimbursement of return airfare to Brazil for the project coordinator, including immediate family members (spouse and children), applicable to researchers residing abroad at the time of project approval

Authors' own elaboration based on CNPq Ordinance No. 1,708 of 9 April 2024 (CNPq, 2024c), as amended by CNPq Ordinance No. 2,565 of 12 December 2025 (CNPq, 2025a).

The second component within the scope of CNPq is Call CNPq/MCTI/FNDCT No. 22/2024 – Knowledge Brazil Program – Support for Network Projects with Brazilian Researchers Abroad (CNPq, 2024b), hereby Network Projects call. This call envisaged funding up to 380 projects led by Brazilian research institutions, whether public or private, in collaboration with Brazilian researchers based abroad (CNPq, 2024d). Applicants were required to hold a doctoral degree and to have a formal affiliation with the host institution in Brazil. The stated objective of this call is to promote knowledge exchange and engagement with Brazilian researchers abroad, with particular emphasis on the development of tools and processes involving artificial intelligence (CNPq, 2024b). The call allocated a total budget of BRL 230 million from the FNDCT. Each project could receive up to BRL 600,000 in funding, including up to BRL 480,000 for capital and operational expenses, and up to BRL 120,000 for travel and per diem expenses (CNPq, 2024b).

The third component of the program operates within FINEP's scope. The Public Call MCTI/FINEP/FNDCT – Economic Subsidy for Innovation (Continuous Flow) – Knowledge Brazil, as described on the FINEP website, aims to “enable the return of Brazilian researchers and/or specialized technical professionals with experience in research, development, and/or technological innovation who are currently residing abroad, to be hired by Brazilian companies” (FINEP, 2024b). The economic subsidy is a non-reimbursable financial resource that functions as a direct incentive for the project (FINEP, n.d.). Eligibility criteria for the economic subsidy include Brazilian companies headquartered in the national territory, engaged in economic activities involving the production or circulation of goods or services for profit, with an annual gross operating revenue exceeding BRL 90 million (FINEP, 2024b).

Eligible costs must “exclusively cover expenses related to the recruitment and professional development, within companies, of researcher(s) and/or specialized technical professional(s) currently residing abroad who will be repatriated” (FINEP, 2024c). Activities may be funded through the following expenditure categories: personnel costs; per diem and travel expenses; and third-party services provided by legal entities (FINEP, 2024c). It should be noted that FINEP's support, through non-reimbursable economic subsidy funding, requires the provision of financial counterpart contributions to the project. In other words, the company must invest a percentage of the total project value, using its own resources, to cover cost items such as construction and installations, permanent equipment, personnel costs, per diem and travel expenses, and third-party services provided by legal entities (FINEP, 2024c).

FINEP is a relevant funding agency involved in other policy initiatives to support and develop Brazil's industrial sector. Another example, in addition to the call mentioned above, is the Nova Indústria Brasil program (NIB) (Brasil, 2026b).

Regarding submitted and approved projects, according to CNPq (2025b), the final results of Attraction and Retention of Talent call received 1,390 project submissions under Line 1, with an approval rate of 40.7% (566 projects), and 136 company submissions under Line 2, with an approval rate of 24.3% (33 projects). In total, 599 projects were approved under this call. Among the fields of knowledge with the highest number of approved projects, the following stand out in descending order: biological sciences (29.3%, 176 projects), exact and earth sciences (23.2%, 139 projects), engineering (12.8%, 77 projects), human sciences (9.0%, 54 projects), and agricultural sciences (8.6%, 52 projects) (CNPq, 2025b). According to CNPq (2025b), this demand implies an investment of BRL 604 million from the FNDCT over a five-year period.

The approved projects were unevenly distributed across the country's regions. The Southeast region accounted for the largest share, representing 50% of approvals, followed by the Northeast (21%), South (18%), Central-West (7%), and North (4%) (CNPq, 2025b). Thus, institutions in the Southeast region predominated in project allocation, a pattern also observed in other funding initiatives, as this region benefits from a more consolidated scientific infrastructure (Guimarães, 2002). However, according to CNPq (2025b), the allocation of projects in other regions (Northeast, Central-West, and North) exceeded the program's target allocation (32% compared to the required 30%). In addition to Brazil, researchers from 34 other countries had projects approved. However, Brazil accounted for the largest share (44%), followed by the United States (12.8%) and Germany (7.3%) (CNPq, 2025b).

The Network Projects call received 980 proposals, of which 640 were approved, totaling BRL 228.5 million over a two-year period (CNPq, 2024e). According to data from the CNPq Demand and Allocation Panel (Brasil, 2026c), the fields of knowledge with the highest number of approved projects include biological sciences (150 projects, 23%), engineering (122 projects, 19%), exact and earth sciences (121 projects, 18%), and health sciences (117 projects, 18%). According to CNPq (2024e), 33.45% of the approved projects (217) will be coordinated by researchers affiliated with institutions located in the North, Northeast, and Central-West regions. This figure exceeds the original target of 30% for these regions.

Table 5 summarizes the projected number of funded projects, the number of submitted proposals, and the number of approved projects under the program.

**Table 5:** Results of calls under the knowledge Brazil program.

Call	Projected number of funded projects	Submitted proposals	Approved projects
Call CNPq/MCTI/FNDCT No. 21/2024 – Knowledge Brazil Program – Attraction and Retention of Talent	1,000	1,526 (52% above target)	599 (40% below target)
Call CNPq/MCTI/FNDCT No. 22/2024 – Knowledge Brazil Program – Support for Network Projects with Brazilian Researchers Abroad	380	980 (157% above target)	640 (68% above target)
Public Call MCTI/FINEP/FNDCT – Economic Subsidy for Innovation – Knowledge Brazil	None^*^	6^*^	2

Authors' own elaboration based on data from CNPq (2025b, 2024e); FINEP (2024a).

^*^Note: Partial results as of December 2025. Continuous flow—submissions remain open until the total value of approved proposals exceeds the allocated budget by up to 30%. Maximum submission deadline: 31 August 2026.

In this context, the number of submissions exceeded the projected number of funded projects in both CNPq calls; however, there were significant differences in the number of approved projects. Accordingly, fewer projects were funded than initially planned under the Attraction and Retention of Talent call, approximately 40% fewer than projected. Conversely, a greater number of projects were approved under the Network Projects call, approximately 68% more than projected.

Regarding investment, the amounts effectively allocated, remained below initial projections. The largest gap is observed in the call under FINEP, which has so far committed only 8% of the allocated budget. However, as this is a continuous-flow call, these figures may change as new proposals are submitted and evaluated. Table 6 summarizes the estimated and allocated investment across all calls.

**Table 6:** Financial resources of the Knowledge Brazil Program (amounts in BRL, 2024–2025).

Call	Estimated investment (A)	Allocated investment (B)	B/A
Call CNPq/MCTI/FNDCT No. 21/2024 – Knowledge Brazil Program – Attraction and Retention of Talent	BRL 825 million^*^	BRL 604 million^*^	73.2%
Call CNPq/MCTI/FNDCT No. 22/2024 – Knowledge Brazil Program – Support for Network Projects with Brazilian Researchers Abroad	BRL 230 million^**^	BRL 228.5 million^**^	99.3%
Public Call MCTI/FINEP/FNDCT – Economic Subsidy for Innovation – Knowledge Brazil	BRL 500 million	BRL 40.1 million^***^	8.0%

*Authors' own elaboration based on data from CNPq (**2025b*, *2024a**,b,e) and FINEP (**2024a**)*. ^*^ For a five-year period. ^**^ For a 2 year period. ^***^ Partial results as of December 2025.

According to CNPq (2025b), the approved projects involve researchers from 35 different countries; however, an analysis of the Lattes CVs identified 37 countries, as shown in Table 7.

**Table 7:** Distribution of researchers by Country.

Country	*n*	%	Country	*n*	%
Brazil	208	39.39%	Saudi Arabia	2	0.38%
USA	64	12.12%	China	2	0.38%
Germany	41	7.77%	Scotland	2	0.38%
France	25	4.73%	Ireland	2	0.38%
Portugal	25	4.73%	Norway	2	0.38%
England	20	3.79%	Poland	2	0.38%
Spain	19	3.60%	Uruguay	2	0.38%
Italy	16	3.03%	Czech Republic	1	0.19%
Canada	15	2.84%	Peru	1	0.19%
Chile	9	1.70%	Wales	1	0.19%
Belgium	9	1.70%	Northern Ireland	1	0.19%
Mexico	6	1.14%	Israel	1	0.19%
Denmark	5	0.95%	Netherlands	1	0.19%
Argentina	5	0.95%	United Arab Emirates	1	0.19%
Austria	4	0.76%	Colombia	1	0.19%
Sweden	4	0.76%	Singapore	1	0.19%
Switzerland	3	0.57%	Qatar	1	0.19%
Australia	3	0.57%	Without information	20	3.79%
South Africa	3	0.57%			

Authors' own elaboration based on data from the CNPq funding panel (CNPq, 2026b) and Lattes CVs.

It was not possible to identify the country of affiliation in 2024 for 20 researchers (3.79%) based on Lattes CV data.

Table 7 also presents the number of researchers and their respective countries of affiliation in 2024. With regard to the distribution of supported researchers, CNPq (2025b) reported that 44% of researchers with approved projects had Brazil as their country of residence, compared with 39.39% based on the analysis of their Lattes CVs. The discrepancy between the percentages reported by CNPq and those derived from the Lattes analysis may be explained by differences in the underlying datasets. The CNPq figures appear to refer to the total number of approved projects (599), whereas the Lattes-based analysis used data on active fellowships from the funding panel (528). Nevertheless, the distribution of researchers across countries remained consistent in both analyses. The most frequent countries of residence were Brazil (39.39%), the United States (12.12%), and Germany (7.77%).

Regarding highest degree attained, 97.73% held Ph.D., 1.52% had completed master's degree and 0.76% had completed or were undertaking undergraduate studies (exclusive to Line 2).

The analysis of information on the year of highest level academic qualification, based on Lattes CV data, indicates a higher frequency of researchers with more recent postgraduate qualifications particularly between 2014 and 2019 (Figure 1). The highest frequency occurred in 2019, when 71 researchers (13.45%) reported having earned one of these degrees (undergraduate, master's, or doctoral).

**Figure 1:**
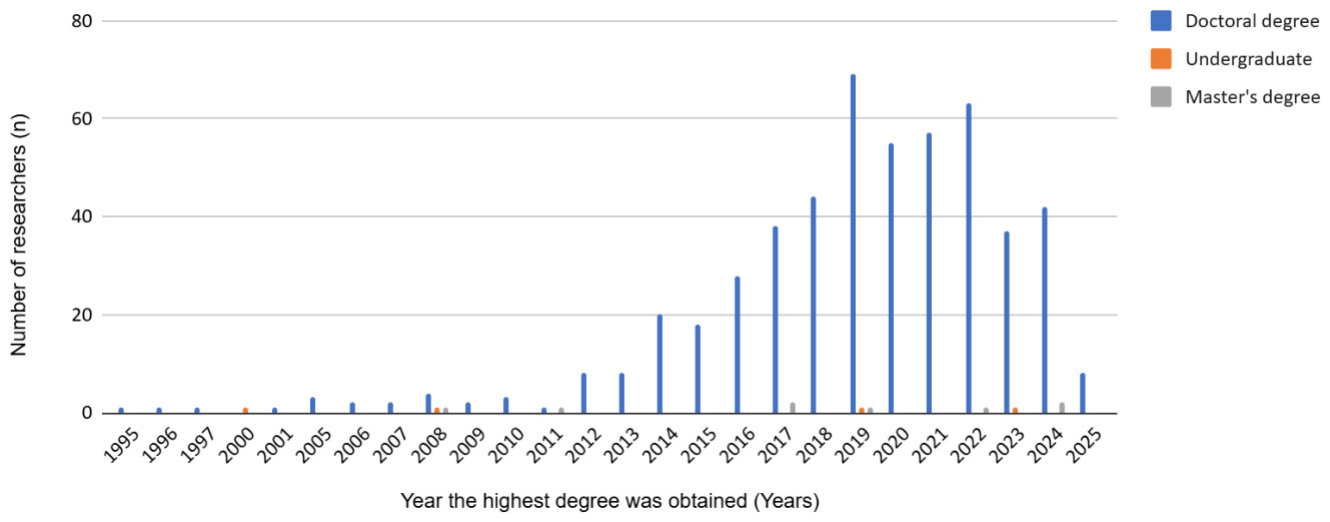
Year the highest degree was obtained Authors' own elaboration based on data from Lattes CVs.

Table 8 presents the academic and professional affiliations in 2024, distinguishing between Postdoctoral researchers, other fixed-term contract and permanent contracts in Brazil and abroad. The analysis of Lattes CV data indicated that 54.55% were post-docs, 29.64% held other fixed-term contracts, that is, they were engaged in temporary contracts, while 15.81% held permanent positions, corresponding to stable academic roles or employment contracts with companies. Regarding permanent contracts, 7.71% of researchers were in Brazil, while 8.10% were abroad. Researchers abroad in fixed-term positions accounted for 51.19% of the total sample, including 31.82% who were postdoctoral researchers. Among those based in Brazil, 33.01% held fixed-term positions, including 22.73% who were postdoctoral researchers.

**Table 8:** Number of researchers by type of contract (fixed-term and permanent).

Type of contract	Brazil (n)	Brazil (%)	Abroad (n)	Abroad (%)	Total (n)	Total (%)
Postdoctoral researchers	115	22.73%	161	31.82%	276	54.55%
Other fixed-term contract	52	10.28%	98	19.37%	150	29.64%
Permanent contract	39	7.71%	41	8.10%	80	15.81%
Total	206	40.71%	300	59.29%	506	100.00%

Authors' own elaboration based on data from Lattes CVs. It was not possible to identify the location (Brazil or abroad) and type of contract (Postdoctoral Researchers, Other fixed-term contract or Permanent contract) for 22 researchers based on Lattes CV data.

Figure 2 presents the start year of the researchers' employment relationships as recorded in 2024 in Lattes CVs. A significant increase in postdoctoral positions is observed starting in 2021.

**Figure 2:**
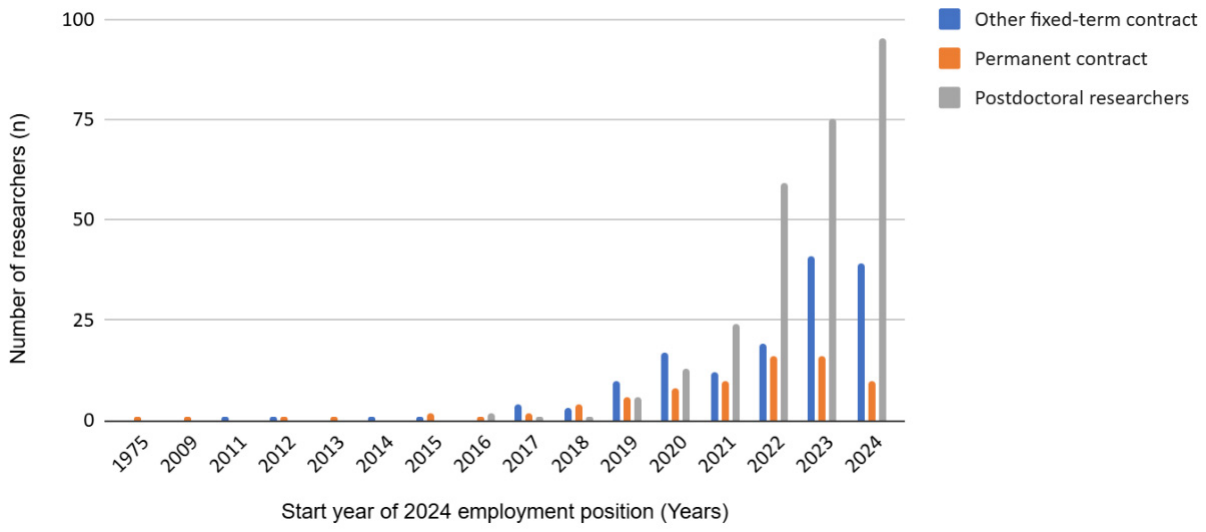
Start year of 2024 employment position. Source: Authors own elaboration based on data from Lattes CVs. Note: It was not possible to identify the start year of 2024 employment position for 22 researchers based on Lattes CV data.

The program faced strong criticism from Brazilian researchers working in the country, as reported in specialized media outlets.

It is important to contextualize the program's announcement, which coincided with a strike across several federal universities, where demands centered on improved salaries and better working and research conditions (Hotta, 2024; Yamashita, 2024).

The criticisms addressed several key issues, primarily focusing on the Attraction and Retention of Talent call. The first concerned the perceived unequal treatment of researchers based in Brazil and those supported by the program. In this regard, critics argued that the fellowship amounts offered by the program were higher than those provided under national funding schemes for researchers in Brazil. In 2025, CNPq fellowship values were BRL 3,100 for doctoral studies and ranged between BRL 5,200 and BRL 5,500 for postdoctoral positions, depending on the modality (CNPq, 2025c). In addition, benefits such as social security contributions and health allowances are not included in fellowships under other CNPq programs.

There were numerous expressions of concern in this regard at the time of the program's announcement. One of the issues raised by Bruno Lupion, a correspondent for Deutsche Welle, was that “the announcement of increased research funding is welcomed by the scientific community; however, some academics argued that, before investing in attracting those abroad, the government should prioritize policies aimed at retaining and supporting PhD holders already in the country.” (Lupion, 2024). It is important to note, however, that according to CNPq (2025b) and an analysis of Lattes CVs, the majority of researchers supported by the program were already in Brazil.

In a similar vein, Nunes (2024) reported that some academics argued that the initiative should be revoked and that priority should instead be given to policies aimed at expanding employment capacity, particularly by increasing the number of academic positions in universities and the number of research institutes. In this context, Professor Yamashita (2024) stated: “The new program addresses short-term issues and does nothing to resolve the structural distortions that lie at the root of the problems it seeks to solve.” For him, the difficulty in securing permanent positions in Brazil, the scarcity of funding, and scientific bureaucracy are the main factors hindering the retention of researchers.

In 2024, the journal Science published an open letter concerning the Knowledge Brazil Program. The publication presented the views of Guedes et al. (2024) regarding perceived shortcomings of the program in relation to Brazil's science and technology system. The authors highlighted that postdoctoral fellowships offer short-term funding without guaranteeing labor rights; that there is a lack of a strategic program to employ newly graduated PhD holders in both academia and the industrial sector; and that, instead of supporting scientists currently unemployed in Brazil, the program prioritized expatriates who may ultimately compete for limited domestic positions with recent graduates (Guedes et al., 2024).

Thus, in the view of these researchers, priority should be given to academics already based in Brazil creating adequate domestic conditions, such as improved research infrastructure and higher-value fellowships for researchers already based in Brazil. However, as noted above, almost half of the projects approved were for researchers in Brazil.

A second issue discussed by experts regarding the Brazilian scientific diaspora concerns the contrast between CNPq's expectation of attracting senior researchers (Jansen, 2024; CNPq, 2025b) and a program design that appears more oriented towards early-career researchers (Carneiro et al., 2024a) or those holding precarious positions abroad. For researchers holding stable positions abroad, the conditions offered may not be sufficiently attractive to encourage them to leave their current institutions and return to Brazil (Lupion, 2024).

Finally, the third issue concerned the sustainability of the program and the retention of researchers after the end of program benefits (Carneiro et al., 2024a). Given that the call was designed to run for 5 years, one concern raised in an interview with Deutsche Welle by a researcher interested in repatriation was whether universities or companies in Brazil would be able to absorb these researchers after this period (Lupion, 2024). The researcher commented:

I think the program is wonderful. But will the Brazilian market actually be able to absorb all the scientists who are planning to return once their fellowships end, or not? There has to be a plan to absorb them after that; otherwise, those who cannot secure positions will end up going abroad again. That is one of my biggest concerns (Lupion, 2024).

In April 2024, CNPq issued a statement on the retention of PhD holders in Brazil, recognizing the role of the state in promoting adequate research conditions and encouraging researchers to remain in the country. To this end, the Council highlighted several strategies, including increased investment in different funding modalities for scientific activities, adjustments to fellowship values, and calls for postdoctoral and doctoral sandwich fellowships (CNPq, 2024f). After announcing the final results of the Knowledge Brazil Program, CNPq stated that it planned to develop initiatives in partnership with MCTI and with support from the FNDCT, including both new programs similar to Knowledge Brazil and programs aimed at retaining young PhD holders in the country (CNPq, 2025b).

In an opinion column published in the newspaper *Correio do Estado*, following the release of the program call results, Fernando Valente Pimentel, Chief Executive Officer of the Brazilian Textile and Apparel Industry Association (ABIT), argued that “Brazil trains talented, creative, and highly skilled individuals, but still faces difficulties in offering them conditions for permanence, fulfillment, and recognition” (Pimentel, 2025). For him, the Knowledge Brazil Program is a source of optimism. In light of the initial results, he describes the program as “a robust initiative that strengthens national science and signals that Brazil seeks—and is able—to attract its talents back” (Pimentel, 2025). However, for this to materialize, the author emphasizes that:

The Knowledge Brazil Program represents progress. However, it must be embedded within a broader and more sustained strategy that integrates policies to value science with a genuine commitment to strengthening public security, healthcare, and education. We need a reliable institutional environment, with stability, fairness, and opportunities (Pimentel, 2025).

In his view, this broader strategy is essential. One reason is the historical instability in funding scientific research in Brazil, which creates uncertainty for long-term projects (Costa and Costa, 2023). Still regarding funding instability, despite the replenishment of the FNDCT and the support of Complementary Law No. 177/2021 (Brasil, 2021), which prohibits the contingency of resources from this fund, the SBPC journal reported in April 2025 that BRL 2.97 billion in reimbursable FNDCT funding was vetoed in the Annual Budget Law (LOA) in order to comply with legal requirements established by another law (SBPC, 2025). Political and economic instabilities often generate uncertainty about the loss of university autonomy, budget cuts, and discourses that devalue science (Carneiro et al., 2022). These factors may affect the sustainability of science and technology policies. At present, new calls are being launched under different configurations. One example, mentioned earlier, is PROFIX-CB. However, given the history of instability, the uncertainty remains.

Thus, according to specialists, it is essential to “foster a more favorable domestic environment for science, making it more attractive both for those already working in the country and for those who may wish to do so” (Carneiro et al., 2024a). In this regard, they emphasize that diaspora policies must be part of a broader set of policies for scientific, technological, social, and cultural development in order to be effective (Carneiro et al., 2024a,b).

The statements discussed above point to structural barriers within the national science and technology system that may challenge the program's long-term sustainability. Particularly important is the lack of a favorable domestic environment for research (Carneiro et al., 2024a). This is related to a broader context marked by the devaluation of science, institutional instability, and a history of unstable funding for science and technology in the country (Pimentel, 2025). In addition, the limited number of labor market positions available to recent PhD graduates affects the conditions for retaining these researchers in Brazil (Yamashita, 2024; Nunes, 2024; Guedes et al., 2024; Lupion, 2024).

With regard to short-term implementation challenges, two situations established in the program calls stand out. The first concerns the requirement of financial counterpart contributions from companies eligible under the FINEP call. The second concerns the novation mechanism: although participation in the project may serve as a form of novation, project proponents cannot have outstanding debts with CNPq or the federal public administration; otherwise, their proposals may be rejected (CNPq, 2024a).

### 4.2 . SWOT analysis

This section presents a diagnostic assessment of the Knowledge Brazil Program using a SWOT analysis, identifying its strengths, weaknesses, opportunities, and threats across internal and external factors.

Based on the analysis of the program design and its initial results, six main strengths of the Knowledge Brazil Program can be identified. The first strength is the more substantive incorporation of the scientific diaspora into the science, technology, and innovation (STI) policy agenda, marked by a shift from viewing it as a loss to recognizing it as an asset to be leveraged. In other words, this represents a reframing of the diaspora as a strategic asset.

The second strength is the program represents an unprecedented policy in Brazil. It is characterized by a rare level of interinstitutional coordination, bringing together multiple government bodies, including the Ministry of Science, Technology and Innovation (MCTI), the Ministry of Foreign Affairs (MRE), CNPq, and FINEP, with funding provided by the FNDCT.

The third strength lies in the program's multi-strategic design. The program is structured into three components:

1. Repatriation component – Attraction and retention of talent in science, technology and innovation institutions (STI institutions) and companies, through incentives to attract master's and doctoral degree holders. This was implemented both through Attraction and Retention of Talent call issued by CNPq and through FINEP's Economic Subsidy call;
2. Linkage component – Support for international collaborative network projects through Network Projects call issued by CNPq;
3. Circulation component – Support for international mobility through a specific budget line within Network Projects call issued by CNPq, providing funding for short-term mobility (airfare and per diem allowances, in accordance with CNPq funding tables for individual grants and short-term fellowships). This enables the circulation of researchers, even in the absence of a dedicated call.

Thus, compared with other internationalization and engagement policies involving the Brazilian scientific diaspora, the program offers a broader design, incorporating the three core policy components: return, linkage, and circulation.

The fourth strength lies in the program's ability to operate as a mechanism of novation. This provision expands the program's target audience and reinforces the signal of a shift in the state's approach towards the diaspora.

The fifth strength is the substantial volume of funding allocated to the program, with the prospect of continuity following the announcement of the PROFIX-CB initiative.

The sixth strength relates to the regional distribution of projects across the national territory. The program achieved the minimum territorial coverage required for the allocation of FNDCT resources, with projects distributed across all regions of the country. In both CNPq calls, the share of projects allocated to the North, Northeast, and Central-West regions exceeded the 30% target.

Eight main weaknesses can be identified. The first weakness is the absence of a formal diagnostic process to inform policy formulation. This suggests a fragility in the program's design, as it is not grounded in a systematic assessment of demand for each policy component, nor in the adoption of best practices from comparable international policies.

The second weakness concerns the asymmetry between the program's axes, which places a clear emphasis on repatriation. As shown in Table 1, contemporary policies for engaging scientific diaspora have increasingly moved towards linkage-based or comprehensive models. In contrast, CNPq allocated a larger share of funding and expected to approve more projects under the Attraction and Retention of Talent call than under the Network Projects call. Moreover, the program's initial name, disseminated by Brazilian diplomatic missions abroad under the Ministry of Foreign Affairs (MRE) from May 2024, already signaled this emphasis on repatriation. However, the final results show that more projects were approved under the Network Projects call than under the Attraction and Retention of Talent call. This may indicate that members of the diaspora are willing to contribute to Brazil without necessarily returning.

The third weakness is that, despite the emphasis on repatriation, the analysis of Lattes CVs revealed that a significant proportion of researchers with approved projects under Attraction and Retention of Talent call were already residing in Brazil in 2024. This suggests ambiguity in the call's target audience and points to a fragility in the “return” narrative, indicating that the call may also have functioned as a mechanism for retention or reintegration of internationally trained researchers.

The fourth weakness relates to the limited institutionalization of circulation strategies. Temporary mobility or short-term visits by researchers appear only as a possibility within Network Projects call, rather than an autonomous policy instrument, as seen, for example, in the Milstein subprogram discussed earlier.

The fifth weakness is the low alignment between the projected targets and the actual implementation of Attraction and Retention of Talent call. The final number of approved projects was 40% lower. This may suggest issues related to operational design, institutional capacity, or eligibility criteria.

The sixth weakness concerns the low initial execution of the FINEP call. This may indicate implementation challenges in aligning the policy instrument with eligible firms, particularly given the requirement for counterpart funding and the focus on larger companies. It should be noted, however, that this is a continuous-flow call, and additional proposals may still be submitted.

The seventh weakness is the uneven regional distribution of projects, with 50% allocated to the Southeast region. This reflects longstanding regional asymmetries, as this region concentrates a higher number of science, technology and innovation institutions (STI institutions) and receives a greater share of funding for research infrastructure (Guimarães, 2002). In addition to federal funding agencies, substantial investment from state-level research foundations also contributes to this concentration and to more favorable research conditions.

The eighth weakness concerns the possibility of using a project under the Knowledge Brazil Program as a novation mechanism. Although this mechanism addresses one of the target groups identified during the formulation of the call, its scope appears limited, as project proponents cannot have pending obligations with CNPq or the federal public administration.

Based on perceptions of policymakers and the academic community regarding the program, five main opportunities related to the context of the Knowledge Brazil Program can be identified.

The first opportunity lies in the recent shift in the federal government's approach, which has prioritized the resumption of investment in science, technology and innovation (STI). This has resulted in the recent replenishment of the FNDCT and a reorientation in the relationship with the scientific diaspora, thereby enabling investment in the program (Lupion, 2024; Pimentel, 2025).

The second opportunity can be observed in an interview with a diaspora researcher reported by Lupion (2024). According to the interviewee, the program offers an opportunity for researchers to fulfill their desire to contribute to the development of research in Brazil, or even to return to the country. In this sense, the program contributes to making the institutional environment more conducive to interaction and to recognizing the scientific diaspora as a strategic asset for the development of STI in the country. The program's results also indicate a willingness among members of the Brazilian scientific diaspora to contribute—either through return or through linkage, given that both calls received more proposals than initially projected.

The third opportunity is the potential for articulation with broader policies on internationalization and industrial innovation. As a program linked to the Ministry of Science, Technology and Innovation (MCTI) and FINEP, it may align with the industrial policy launched by the federal government in 2024, the Nova Indústria Brasil (NIB) program, by attracting and retaining highly qualified human resources to support industrial and technological innovation. This reinforces the relevance of the program's business-oriented component, primarily coordinated by FINEP and also highlights the need for coherence among industrial policy, firms' absorptive capacity and diaspora engagement policies.

The fourth opportunity, highlighted by Carneiro et al. (2024a), is that the program may offer a pathway for return and integration into universities or companies for early-career researchers or those holding precarious positions abroad. By offering more attractive benefits compared with those available domestically, the program creates incentives to integrating these professionals into the national scientific system. The analysis of Lattes data showed that most researchers supported under the Attraction and Retention of Talent call were postdoctoral researchers or held other fixed-term positions. Additionally, the work experience at the institutions under the fellowship can increase their chances in future recruitment processes.

The fifth opportunity is based on the performance of the call Network Projects, which exceeded its original target. This result suggests a window of opportunity for policies less centered on permanent return and more focused on linkage. It may also indicate latent demand for international collaboration and scientific reintegration.

Four main external threats can be identified in the external environment. The first threat concerns the sustainability and long-term stability of funding, which are essential for advancing science and technology but have not been consistently ensured in Brazil's recent past. Even with the recent replenishment of the FNDCT, issues related to the execution of resources persist. Combined with the historical instability in the use of STI funding instruments in Brazil, as noted by Costa and Costa (2023), this can be considered the first major threat to the program.

The second threat, highlighted by Carneiro et al. (2024a) and Lupion (2024), concerns the lack of predictability regarding whether academia or the labor market will be able to absorb researchers after the program's completion.

The third threat concerns the negative perception of the program among Brazilian researchers, which may generate tensions between program fellows and locally based faculty Hotta (2024); Yamashita (2024). As discussed, many researchers argued that the resources allocated to the Knowledge Brazil Program should instead be prioritized for domestic improvements. This reflects a moralized framing of the diaspora (“supporting those who left” vs “supporting those who stayed”), which goes beyond isolated opinions and may threaten the program's political legitimacy. It is important to note, however, that although FNDCT resources may be used to finance university infrastructure, funding for academic salaries, postgraduate fellowships, and the operating costs of federal universities derive from different budgetary sources within ministerial allocations.

In this regard, a fourth threat can be systematized based on the views expressed by Lupion (2024), Pimentel (2025), Carneiro et al. (2024a),b), Yamashita (2024), Nunes (2024), and Guedes et al. (2024) which emphasize the need to address the conditions of the domestic research environment. This includes assessing national needs in terms of research infrastructure, adequate funding, career satisfaction among researchers, the capacity to conduct research and innovation within firms operating in the country, and the ability of universities and companies to absorb researchers, among other factors. These concerns relate to the need for increased investment and resources, as well as for an attractive, secure, and sustainable environment for conducting scientific research, with opportunities available to researchers across career stages. In this sense, the threat lies in the mismatch between the program and the structural constraints of the national STI system. The central critique is not limited to the future absorption of program fellows, but rather to the risk that the program may be expected to address structural issues beyond its scope, such as career pathways, the availability of stable positions, research infrastructure, and the baseline funding of universities.

The SWOT analysis is synthesized in Table 9.

**Table 9:** SWOT Analysis of the knowledge Brazil program.

Environment	Strengths	Weaknesses
Internal Environment	•Reframing of the scientific diaspora as a strategic asset. •Unprecedented national policy for engaging with the diaspora •Interinstitutional coordination between MCTI, CNPq, FINEP, and MRE. •Broad policy design (repatriation, linkage through networks, and indirect circulation) •Significant volume of financial resources mobilized. •Possibility of using the program as a mechanism of novation •Nationwide coverage, exceeding the minimum regional allocation target	•Absence of a formal and systematic diagnostic process in policy formulation. •Strong emphasis on repatriation in discourse and initial design, despite the stronger performance of the network component in terms of proposal submissions. •Ambiguity in the concept of “repatriation” (great part of the approved researchers based in Brazil). •Circulation appears only indirectly. •Low alignment between targets and implementation in the Attraction and Retention of Talent call. •Very low initial execution of the FINEP component •Persistent regional concentration in the Southeast •Limited application of the debt novation mechanism for scholarship recipients with outstanding obligations to the National Treasury.
	Opportunities	Threats
External Environment	•Recent replenishment of the FNDCT and expansion of federal investment capacity in STI. •An institutional environment more conducive to engagement with the scientific diaspora. •Potential alignment with internationalization policies and the industrial policy. •Opportunity for return and integration of early-career researchers or those in precarious positions abroad. •High demand for network-based collaboration, indicating scope for strengthening brain linkage and circulation strategies.	•Historical instability in STI funding instruments in Brazil. •Limited capacity for stable absorption of researchers after the program's completion. •Risk of reduced political legitimacy of the program, particularly in a context of resource constraints and perceptions of preferential treatment towards the diaspora. •Potential misalignment between the program's ambitions and the domestic system's capacity to provide career pathways, infrastructure, and institutional continuity.

Authors' own elaboration.

## 6 Conclusions

This article analyzed the design and early implementation of the Knowledge Brazil Program in order to identify the strategies prioritized by the Brazilian state for engaging with its scientific diaspora. It sought to understand how this policy is positioned within the broader debate on scientific diaspora engagement, particularly regarding strategies of repatriation, linkage, circulation, and talent retention.

The findings of SWOT analysis indicate that the program has an ambitious institutional design, supported by significant financial investment and strategic axes aligned with the framing of the diaspora as a strategic asset for Brazilian science. However, the program remains conditioned by issues of predictability and continuity, which raise concerns about its long-term sustainability and reinforce the need for sustained engagement policies. Brazil was relatively slow to recognize its diaspora as a strategic asset, resulting in a gap in the development of consistent and sustainable public policies for this group. As highlighted by Carneiro et al. (2024b), diaspora engagement policies should not be viewed as an end in themselves; rather, they must recognize the capacities, resources, and needs on both sides, those of the diaspora and of the domestic environment, in order to be sustainable within a context of mutual trust.

Furthermore, investment in international collaboration through engagement with diaspora networks can be highly advantageous for the country, particularly in an increasingly internationalized scientific landscape (Balbachevsky and Couto e Silva, 2011; Tejada, 2012). Engagement with the international environment and with expatriate researchers can create opportunities for the exchange of materials, methods, resources, and expertise, thereby generating mutual benefits for both domestic and international contexts (Balbachevsky and Couto e Silva, 2011; Tejada, 2012; Meyer and Wattiaux, 2006).

As this is a recent policy, the study has limitations. Implementation outcomes are still preliminary, and the analysis of the program's design relied primarily on secondary data sources, without triangulation with primary data. In addition, the study is limited by the absence of direct stakeholder and program beneficiary participation in the analysis. We recognize that the lack of participation by these internal policy actors may result in a less comprehensive mapping of operational bottlenecks and of the relationship between the program theory and its implementation in practice.

The analysis to date raises several avenues for future research. First, further investigation is needed into the misalignment between targets and implementation in the Attraction and Retention of Talent call. Second, long-term studies are required to assess institutional retention and the consolidation of international scientific networks. Third, monitoring and evaluation studies of the program would be particularly valuable, especially if they included the participation of stakeholders and direct beneficiaries. Such studies could help address some of the limitations of the present analysis and assess the program's long-term results.

Finally, it is concluded that the Knowledge Brazil Program represents a relevant policy in the debate on mobility and engagement with the scientific diaspora. However, its effectiveness will depend on its integration into a broader, structural, and sustainable policy aimed at strengthening the domestic scientific environment. By highlighting this interdependence and by combining consolidated theories in the field of diaspora engagement policies with a systematic methodological approach, this article contributes to the analysis of such policies and supports stakeholder decision-making regarding their formulation, implementation, and maintenance.

## Data Availability

The original contributions presented in the study are included in the article and in the Supplementary material. Further inquiries can be directed to the corresponding author.
