# Supplementary material for: Between linkage and return: the engagement strategies of the Knowledge Brazil Program for the scientific diaspora
Source: Front Res Metr Anal. 2026 Sep 17;11:1854887. doi: 10.3389/frma.2026.1854887 (PMC13627378; doi:10.3389/frma.2026.1854887)
Supplement: Supplementary file 1 [file Table_2.docx]

The prompt below was used in ChatGPT Plus to revise the manuscript. After that, all material were properly revised by us.

**Prompt:** The following text is part of an academic article to be published in English. Translate the passages that are in Portuguese and suggest improvements to the text already written in English so that it becomes more fluent, clearer, and more academic in tone.
